# Predictive Value of Delta-Radiomics Texture Features in 0.35 Tesla Magnetic Resonance Setup Images Acquired During Stereotactic Ablative Radiotherapy of Pancreatic Cancer

**DOI:** 10.3389/fonc.2022.807725

**Published:** 2022-04-19

**Authors:** Garrett Simpson, William Jin, Benjamin Spieler, Lorraine Portelance, Eric Mellon, Deukwoo Kwon, John C. Ford, Nesrin Dogan

**Affiliations:** Radiation Oncology, Miller School of Medicine, University of Miami, Miami, FL, United States

**Keywords:** delta-radiomics, texture analysis, pancreas cancer, MRI, low field (0.35 T)

## Abstract

**Purpose:**

The purpose of this work is to explore delta-radiomics texture features for predicting response using setup images of pancreatic cancer patients treated with magnetic resonance image guided (MRI-guided) stereotactic ablative radiotherapy (SBRT).

**Methods:**

The total biological effective dose (BED) was calculated for 30 patients treated with MRI-guided SBRT that delivered physical doses of 30–60 Gy in three to five fractions. Texture features were then binned into groups based upon BED per fraction by dividing BED by the number of fractions. Delta-radiomics texture features were calculated after delivery of 20 Gy BED (BED20 features) and 40 Gy BED (BED40 features). A random forest (RF) model was constructed using BED20 and then BED40 features to predict binary outcome. During model training, the Gini Index, a measure of a variable’s importance for accurate prediction, was calculated for all features, and the two features that ranked the highest were selected for internal validation. The two features selected from each bin were used in a bootstrapped logistic regression model to predict response and performance quantified using the area under the receiver operating characteristic curve (AUC). This process was an internal validation analysis.

**Results:**

After RF model training, the Gini Index was highest for gray-level co-occurrence matrix-based (GLCM) sum average, and neighborhood gray tone difference matrix-based (NGTDM) busyness for BED20 features and gray-level size zone matrix-based (GLSZM) large zones low gray-level emphasis and gray-level run length matrix-based (GLRLM) run percentage was selected from the BED40-based features. The mean AUC obtained using the two BED20 features was AUC = 0.845 with the 2.5 percentile and 97.5 percentile values ranging from 0.794 to 0.856. Internal validation of the BED40 delta-radiomics features resulted in a mean AUC = 0.567 with a 2.5 and 97.5 percentile range of 0.502–0.675.

**Conclusion:**

Early changes in treatment quantified with the BED20 delta-radiomics texture features in low field images acquired during MRI-guided SBRT demonstrated better performance in internal validation than features calculated later in treatment. Further analysis of delta-radiomics texture analysis in low field MRI is warranted.

## Introduction

Radiomic analysis is the extraction of quantitative image descriptors from radiographic images. Radiomic texture features quantify image gray-level spatial relationships that are hypothesized to represent a tumor’s underlying microenvironment (1). Tumor heterogeneity presents a major obstacle for personalized medicine and effective treatment, and quantification of the heterogeneity could provide valuable insight (2, 3). Utilization of radiomic features is attractive, as it can provide a non-invasive patient-specific biomarker based on routinely acquired images (4–6). Interest in radiomic texture features intensifies, as they are found to have utility in predictive modeling, support clinical decision-making, and are linked with phenotypic and genetic profiles of various cancers (7–9).

Application of radiomic analysis in pancreatic cancer patients is of particular interest due to the poor treatment outcomes. The incidence of pancreatic cancer continues to increase with a low 5-year overall survival of approximately 9% and is predicted to be one of the top 5 contributors of cancer deaths among men and women in the United States in 2021 (10). Surgery offers patients with pancreatic ductal adenocarcinoma (PDAC) the best chance of long-term survival, but only a minority of patients, 15%–20%, qualify for resection (11). Patients diagnosed as having borderline resectable or unresectable disease generally receive upfront chemotherapy (nCRT) followed by stereotactic body radiation therapy (SBRT) (12–14). This regime can induce changes in the gross disease and allow patients to qualify for surgery. Furthermore, SBRT has improved complete pathological response rates from 2.5% to 7.8% (15). Real-time disease monitoring *via* radiomic analysis may provide valuable personalized information that could lead to beneficial adaptation of the patient care path and improved outcome.

The requirement and desire for better visualization of tumor target and organs at risk locations in patient populations like PDAC patients during therapy have driven the development of new image guidance technology. Magnetic resonance imaging (MRI)-guided radiotherapy units provide an ideal platform for treatment of pancreas SBRT patients because of the ability to acquire high-quality images. MR images have improved soft tissue contrast compared to traditional X-ray guidance modalities and allow for safe dose escalation and avoidance of critical organs in proximity to the target. The daily MRIs acquired prior to each treatment are a by-product of the patient care path but may contain valuable information about patient disease over the course of treatment. These daily images provide an opportunity for constructing models predictive of treatment response for PDAC patients using radiomic analysis. A previous work has indicated that the low field strength MR images provide an adequate base for texture analysis (16). Furthermore, a longitudinal image series may bring the opportunity to monitor tumor microenvironment changes during treatment.

Using the average radiomic feature value throughout the treatment demonstrated possible prognostic utility, but consideration of the time that a prediction is made is important (17). A prediction made too late in the treatment is not ideal because the knowledge provided is unlikely to positively impact a patient’s care. Interest and application of radiomic texture features has shifted towards delta-radiomics, which considers changes in radiomic feature values in response during treatment. Delta-radiomics may provide valuable patient-specific outcome prediction with multiple fractions remaining. Indication of disease response prior to the end of radiotherapy treatment could provide valuable information for physicians to consider options for the remainder of the treatment. A limited number of studies have explored delta-radiomic analysis on diagnostic images acquired prior, periodically during, or after treatment (18–21). Nasief et al. used delta-radiomics texture features calculated as the change relative to pre-fraction values extracted from CT images of pancreatic cancer patients to predict treatment outcome (22). High-contrast diagnostic images with better resolution are generally considered ideal for radiomics analysis; however, the limited number of images could limit exploration of delta-radiomics. The increased reliance on imaging in radiation oncology is producing longitudinal image series. The indication of prognostic performance of delta-radiomics features extracted from cone beam computed tomography images was encouraging and has led to further interest in using setup images from other modalities (23). MRI-guided units produce daily setup images for each patient with previous work from our group, indicating that these images may provide a basis for radiomics analysis (24). Radiomics analysis of low field MRIs has gained popularity, but delta-radiomics analysis of images in SBRT for response prediction in PDAC patients is still evolving. The purpose of this study is to use low field strength MR images acquired prior to each daily treatment of PDAC patients treated with MRI-guided SBRT to evaluate the feasibility of using delta-radiomic features for predicting patient response.

## Materials and Methods

### Patient Database

To qualify, patients should have biopsy-confirmed PDAC, including locally advanced or borderline resectable disease. Additionally, each patient should have completed chemotherapy prior to MR-guided SBRT. Patients’ individual treatment schedules were used to group fractions with similar amounts of delivered dose together. The total biological effective dose (BED) was calculated for each patient using an alpha/beta ratio = 10 (25). Patients were treated with three to five fractions (1–2 weeks), with a total BED ranging from 54.8 to 132 Gy with a median of 100 Gy (physical dose ranging from 30 to 60 Gy and a median of 50 Gy). Treatments were planned and adapted as needed using an isotoxic approach to escalate the dose to the target until organs at risk dose constraints neared their maximums (26).

Patient treatment response was based upon post-surgery pathology or post-treatment imaging studies. Treatment response for patients who had undergone curative-intent resection following SBRT utilized tumor response grading with the College of American Pathologists (TRG-CAP). Response for patients with imaging studies was determined according to modified response evaluation criteria in solid tumors (mRECIST 1.1) (27, 28). A previously used binary response classification scheme was used to assign each patient to the responder (RS) or non-responder (NR) category (17). Response for patients able to undergo resection was determined using TRG-CAP. TRG-CAP scores ≤2 were considered responders and a score = 3 as NR. For patients who did not qualify for curative resection, follow-up imaging using mRECIST 1.1 criteria was utilized and was determined using dynamic CT, MRI, or PET images acquired within 1–3 months after SBRT.

### Daily Setup Images and Volume Delineation

Daily images were acquired on the 0.35-T split-bore MRI-guided radiation treatment unit prior to each daily treatment (MRIdian, ViewRay Inc., Cleveland, OH). Images utilized for this work were acquired using the clinical pulse sequence. The sequence is a true fast imaging with balanced steady-state free procession pulse sequence (bSSFP). bSSFP is a popular choice for MR-guided RT systems because of the short acquisition time needed for volumetric image acquisitions, high signal contrast in soft tissue, insensitivity to motion, and minimal spatial integrity perturbation. The images contained a blend of T1- and T2-weighted visual characteristics (29). Clinical setup images were acquired with 1.5 × 1.5 × 3.0 mm^3^ voxel dimensions. Three variations of the clinical bSSFP sequence were included and can be seen in **Table 1**. The inclusion of multiple variations of the same pulse sequence was deemed acceptable because of identical acquisition times, identical voxel sizes, identical flip angles, and similar repetition time (TR) and echo time (TE) (30). Any difference in parameters should have a minimal impact on feature value. Images of patients with arms above their heads were acquired using an MR-compatible board and surface coil. After patients finished treatment, the images were exported to MIM Maestro v6.5.5 (MIM, Software, Cleveland, OH) for contouring. The gross tumor volume (GTV) was contoured on each daily setup image by a radiation oncologist with expertise in PDAC.

**Table 1:** The three versions of the bSSFP pulse sequence employed by the MR system for alignment of patients prior to radiotherapy delivery.

Acquisition Parameter	Image Pulse Sequences		
	Clinical A	Clinical B	Clinical C
TR/TE (ms)	3.00/1.27	3.33/1.43	3.36/1.44
Bandwidth (Hz/pixel)	604	537	534
Field of view (mm)	540 × 465 × 432	400 × 400 × 432	350 × 350 × 432
Matrix size	360 × 360 × 144	266 × 266 × 144	234 × 234 × 144

All pulse sequences have identical flip angles and voxel dimensions.

### Radiomic Texture Feature Extraction

After contouring, the GTVs were exported from MIM to be processed in MATLAB 2020b (The MathWorks, Inc., Natick, MA). Prior to radiomic texture feature calculation, the intensity range of each GTV was normalized by limiting the dynamic range using the methods of Collewet et al., the “± 3σ” method (S4) (31). The GTVs were then quantized to 64 gray levels with the histogram equalization method using a combination of in-house programs and publicly available codes (24, 32). Radiomic texture features belonging to gray-level co-occurrence matrix-based (GLCM, IBSI aggregation code IAZD), gray-level size zone matrix-based (GLSZM, IBSI aggregation code KOBO), gray-level run length matrix-based (GLRLM, IBSI aggregation code IAZD), and neighborhood gray tone difference matrix-based (NGTDM, IBSI aggregation code KOBO) were calculated from each daily setup image (33–39). Details regarding aggregation codes and feature names for the 39 radiomic texture features are displayed in **Table 2**. Five radiomic features were modified in accordance with that of Fave et al. to remove some intrinsic volume dependence (40).

**Table 2:** Second-order radiomic texture features serving as the basis for the delta-radiomics texture features.

Matrix encoding class (IBSI/aggregation code)	Radiomic texture feature (IBSI code)
GLCM (LFYI/IAZD)	Contrast (ACUI)
	Dissimilarity (8S9J)
	Homogeneity (IB1Z)
	Correlation (NI2N)
	Energy (8ZQL)*
	Variance (UR99)
	Entropy (TU9B)
	Sum average (ZGXS)
GLRLM (TPOI/IAZD)	Short run emphasis (220V)
	Long run emphasis (W4KF)
	Gray-level non-uniformity (R5YN)*
	Run length non-uniformity (W92Y)*
	Run percentage (9ZK5)
	Low gray-level run emphasis (V3SW)
	High gray-level run emphasis (G3QZ)
	Short run low gray-level emphasis (HTZT)
	Short run high gray-level emphasis (GD3A)
	Long run low gray-level emphasis (IVPO)
	Long run high gray-level emphasis (3KUM)
	Gray-level variance (8CE5)
	Run length variance (8CE5)
GLSZM (9SAK/KOBO)	Small zone emphasis (5QRC)
	Large zone emphasis (48P8)
	Gray-level non-uniformity (JNSA)
	Zone-size non-uniformity (4JP3)
	Zone percentage (P30P)
	Low gray-level zone emphasis (XMSY)
	High gray-level zone emphasis (5GN9)
	Small zone low gray-level emphasis (5RAI)
	Small zone high gray-level emphasis (HW1V)
	Large zone low gray-level emphasis (YH51)
	Large zone high gray-level emphasis (J17V)
	Gray-level variance (BYLV)
	Zone-size variance (BYLV)
NGTDM (IPET/KOBO)	Coarseness (QCDE)*
	Contrast (65HE)
	Busyness (NQ30)*
	Complexity (HDEZ)
	Strength (1X9X)

GLCM, gray-level co-occurrence matrix; GLRLM, gray-level run length matrix; GLSZM, gray-level size zone matrix; NGTDM, neighborhood gray tone difference matrix; IBSI, is the Image Biomarker Standardization Initiative. Features marked with * were IBSI features modified to decrease volume independence (39).

All radiomic texture features were calculated *via* the 3-D definitions using the 26-nearest neighbor voxels. GLCM-based features were calculated using a voxel displacement of one. The GLCM is populated by calculating the probability of gray-level intensities occurring together within a given region. While the GLCM records two voxel intensities, GLSZM and GLRLM record connected isotone zones and regions, respectively. Coordinates in the GLSZM correspond to the probability of a zone of a gray level of a size occurring with the region of interest. Similarly, the GLRLM encodes the probability of a gray level occurring in a connected run of a certain size. The NGTDM records the average difference between each gray level and the voxels surrounding it. Each radiomic texture feature sums over the probabilities within the class matrix. The result is a single descriptive number for each feature emphasizing different characteristics of gray-level intensity relationships within the image.

Two sets of delta-radiomics texture features were calculated for each patient using the BED/fraction to group features calculated from different time points into similar delivered dose regions. The total BED was divided by the number of fractions and serves to represent features as a function of time and of dose. Delta-radiomics features were calculated by taking the difference in feature values between pre-radiation images (fraction 1 setup images) and then after a certain amount of dose was delivered. Specifically, for this patient cohort, the following dose bins of 20 and 40 Gy were used and named BED20 and BED40, respectively. BED20 features were calculated after the delivery of 20–30 Gy and BED40 features after 40 Gy.

### Radiomic Texture Feature Selection and Analysis

A supervised machine learning approach, random forest (RF), was used to explore the data and select potentially predictive features using a two-step process (41). RF is an ensemble method that creates many weaker learners called decision trees by splitting the training data and combining many decision trees into a forest. Combining the weak learners allows the model to overcome variability in the data and is considered a strong learner (42). The first step of the process was to select potentially prognostic values using the Gini Index that quantifies how important each feature is for accurate predictions during RF model training (43). An RF model was trained using BED20 or BED40 delta-radiomics texture features to predict binary patient outcome. The Gini Index, a measure of variable importance for predictive accuracy, was obtained during model training by calculating the out-of-bag error using bootstrapped datasets from building 500 trees (weak learners) in R (R Foundation for Statistical Computing, Vienna, Austria) (42). The top 2 ranking features were evaluated using an internal validation approach to avoid overfitting due to the limited sample size of patients. A bootstrapped logistic regression model was used to evaluate the pre-selected delta-radiomic features by bootstrapping the data 1,000 times and selecting two-thirds (20 patients at a time) to construct the logistic regression. The resulting model was used to predict the outcome for all patients and to calculate area under the receiver operating characteristic curve (AUC) for each iteration. The numbers reported are internal validation AUCs based upon the 2.5 and 97.5 percentile values, and the mean value of the 1,000 iterations.

## Results

### Patient Database

A total of 30 patients were included for analysis. The RS group consisted of 11 patients with complete or partial responses. The patients with progressive and stable diseases were included in the NR group and totaled 19. **Table 3** displays each patient’s response along with the total BED and delivered BED/fraction.

**Table 3:** Characteristics of patients included for delta-radiomics texture analysis.

Patient Number	Response	Total BED	BED/Fx	BED20	BED40
1	RS	72	14.4	Fx1–Fx3	Fx1–Fx4
2	NR	59.5	11.9	Fx1–Fx3	Fx1–Fx5
3	NR	72	14.4	Fx1–Fx3	Fx1–Fx4
4	NR	100	20	Fx1–Fx2	Fx1–Fx3
5	NR	59.5	11.9	Fx1–Fx3	Fx1–Fx5
6	RS	54.8	11.0	Fx1–Fx3	Fx1–Fx5
7	RS	72	14.4	Fx1–Fx3	Fx1–Fx4
8	NR	72	14.4	Fx1–Fx3	Fx1–Fx4
9	NR	72	14.4	Fx1–Fx3	Fx1–Fx4
10	RS	59.5	11.9	Fx1 - Fx3	Fx1–Fx5
11	NR	100	20.0	Fx1–Fx2	Fx1–Fx3
12	NR	100	20.0	Fx1–Fx2	Fx1–Fx3
13	RS	100	20.0	Fx1–Fx2	Fx1–Fx3
14	RS	132	26.4	Fx1–Fx2	Fx1–Fx3
15	RS	100	20.0	Fx1–Fx2	Fx1–Fx3
16	RS	100	20.0	Fx1–Fx2	Fx1–Fx3
17	NR	100	20.0	Fx1–Fx2	Fx1–Fx3
18	RS	61.5	12.3	Fx1–Fx3	Fx1–Fx5
19	RS	100	20.0	Fx1–Fx2	Fx1–Fx3
20	RS	100	20.0	Fx1–Fx2	Fx1–Fx3
21	NR	100	20.0	Fx1–Fx2	Fx1–Fx3
22	NR	100	20.0	Fx1–Fx2	Fx1–Fx3
23	NR	100	20.0	Fx1–Fx2	Fx1–Fx3
24	NR	100	20.0	Fx1–Fx2	Fx1–Fx3
25	NR	100	20.0	Fx1–Fx2	Fx1–Fx3
26	NR	100	20.0	Fx1–Fx2	Fx1–Fx3
27	NR	100	20.0	Fx1–Fx2	Fx1–Fx3
28	NR	100	20.0	Fx1–Fx2	Fx1–Fx3
29	NR	100	20.0	Fx1–Fx2	Fx1–Fx3
30	NR	72	14.4	Fx1–Fx3	Fx1–Fx4

The second column contains the binary response classification for each patient, the third column is the total BED for the entire treatment, and the column “BED/Fx” is the total BED divided equally between the number of fractions (in Gy). The final two columns contain the fractions used to calculate the difference corresponding to the binning scheme for delivered dose for each type of delta-radiomics texture features.

### Feature Selection and Analysis

**Figure 1:**
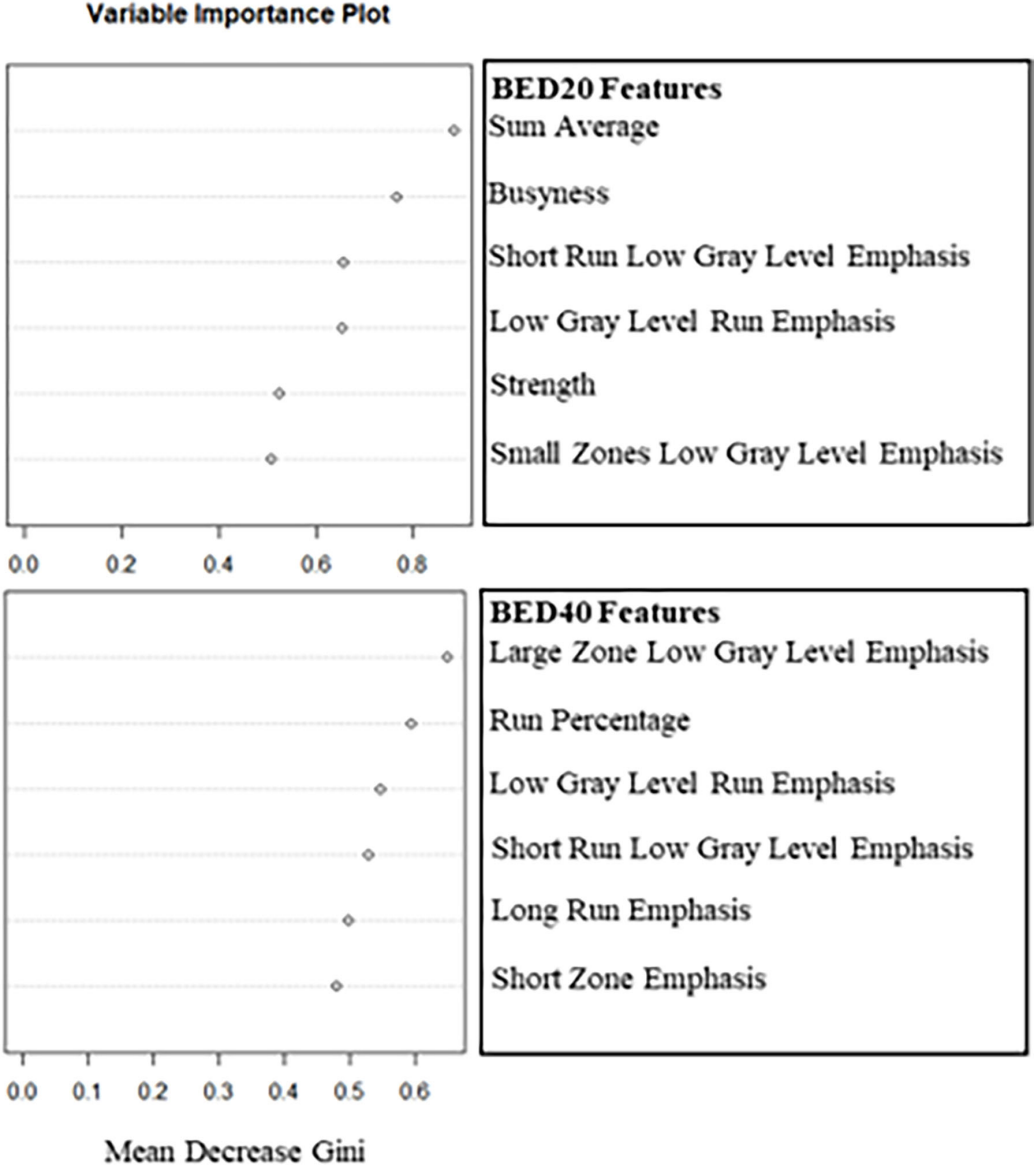
Plots of the mean decrease in the Gini Index used to select the two most important deltaradiomics texture features after training the RF to predict binary outcome. The top plot consists of the BED20-based radiomics texture features and the bottom contains the BED40-based features.

The delta-radiomics texture features selected by each model as important for prediction during model training can be seen in **Figure 1**. Based on the Gini Index, GLCM sum average and NGTDM busyness were selected for further analysis during RF model training for BED20-based delta-radiomics texture features, as seen in the top half of **Figure 1**. The higher the value of the Gini Index is, the more important the feature is for model predictive accuracy. The other features included were ranked lower based on the Gini Index, indicating less predictive importance. For the BED40 delta-radiomics texture features (bottom of **Figure 1**), GLSZM large zones low gray-level emphasis and GLRLM run percentage were selected as most important for model prediction accuracy. The Gini Index values for the top 6 features were ranked closer to each other than the BED20-based features. The results of the bootstrapped logistic regression modeling using the respective features are displayed in **Table 4**. Delta-radiomics features extracted early in treatment, BED20-based features, achieved higher AUCs than the BED40 features. The mean internal validation AUC obtained during the bootstrapped logistic regression using the BED20 features with the percentile range from 2.5% to 97.5% was AUC = 0.845 [0.794–0.856]. The mean AUC for the BED40 delta-radiomics was AUC = 0.567 [0.502–0.675].

**Table 4:** The left column contains row labels for the BED20 (middle column) and the BED40 (right column) for relevant radiomic texture features selected by the Gini Index and their internal validation AUCs obtained from bootstrapped logistic regression analysis.

Delta-radiomics feature type:	BED20	BED40
Feature 1	GLCM sum average	Low gray-level run emphasis
Feature 2	Large zones low gray level-emphasis	Run percentage
Mean AUC	0.845	0.567
2.5% AUC value	0.794	0.502
97.5% AUC value	0.856	0.675

**Figure 2:**
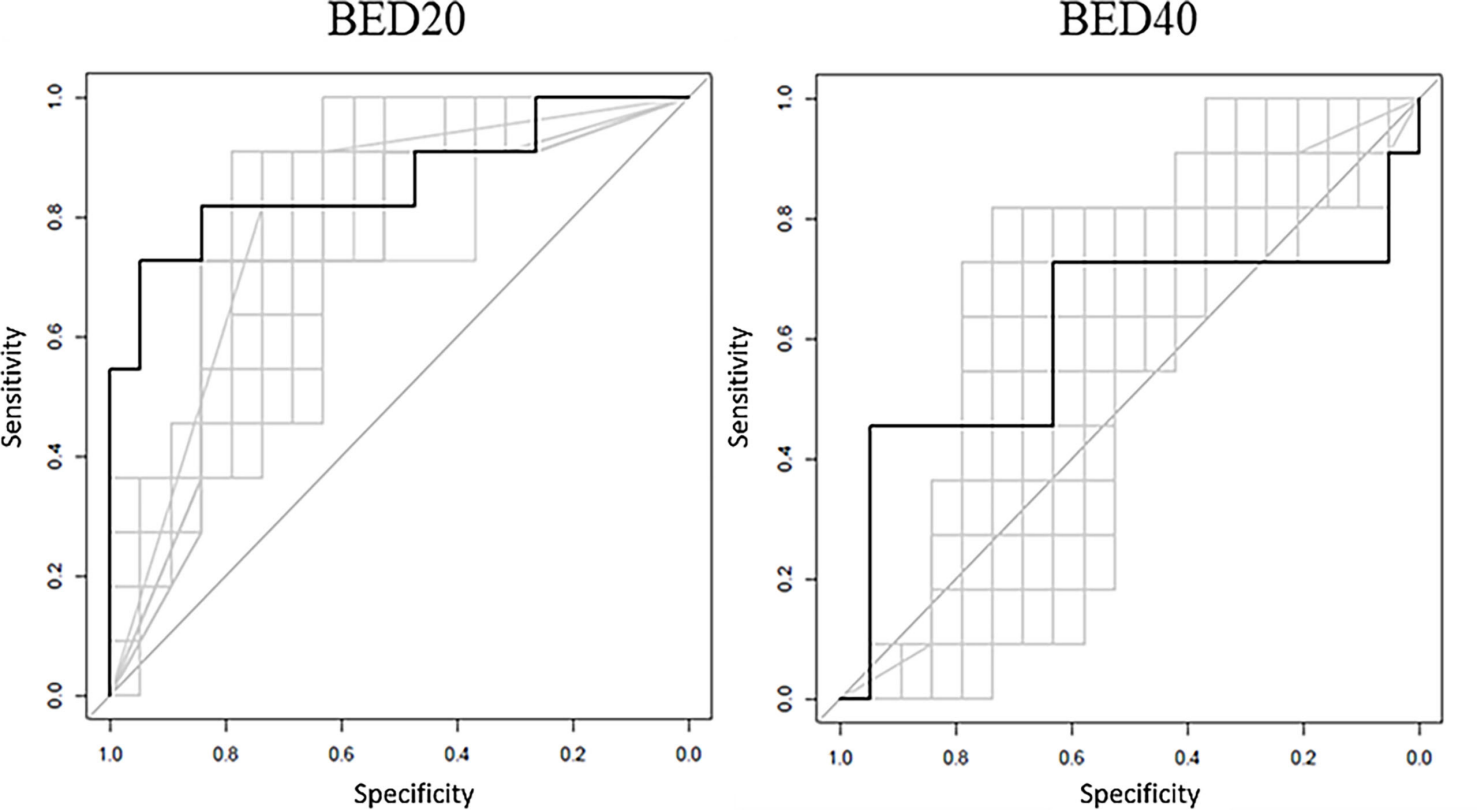
The black ROC curves represent the RF model estimates based on model training. The RF model AUC = 0.876 for BED20 and AUC = 0.601 for the BED40 RF model. The gray curves represent the returned bootstrapped logistic regression produced using the top two features, with an average AUC = 0.845 for BED20 and AUC = 0.567 for BED40-based features.

**Figure 3:**
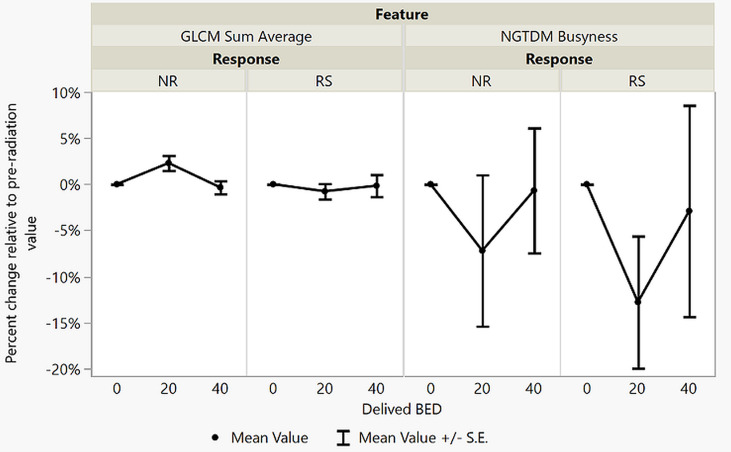
The values of the top two performing texture features in terms of internal validation AUC from the BED20 model. Y-axes are the percent change relative to the pre-RT (0 Gy) values graphed at each point of 20 Gy and 40 Gy. The plots display the mean percent change in each feature value, the points, the ranges represent the range of the standard error from the means.

The ROCs calculated during the logistic regression analysis and the RF model ROC estimates are displayed in **Figure 2**. The values of the top 2 BED20 delta-radiomics texture features are displayed in **Figure 3**, with the Y-axis as the mean radiomic texture feature values and the X-axis containing the bin for delivered dose that the feature values were assigned to for RS and NR patients for the two most predictive delta-radiomic texture features selected at the BED20 timepoint. The features are graphed as the percent change from pre-RT values for each feature and group of patients. The RS group generally had small increases in GLCM sum average values (negative percent change from pre-RT values) from pre-radiation features to BED20, then values return to near pre-treatment levels by the BED40 timepoint. Most of the NR group features decreased at BED20 (positive percent change) and returned to approximately pre-radiation levels also. NGTDM busyness increased from pre-radiation values to BED20 for both RS and NR. While the RS group’s features had little change between BED20 and BED40, the NR group’s features continued to increase in the same period.

## Discussion

Delta-radiomics using patient setup imaging was initially explored by Fave et al. using CBCT images of lung patients (23). The concept of monitoring changes in disease *via* radiomic texture features induced by treatment is beginning to take hold with the increased reliance of imaging in radiation oncology. Jeon et al. calculated delta-radiomics texture features from T2-weighted diagnostic MR images acquired before and after preoperative chemoradiotherapy in locally advanced rectal cancer patients (18). The region of interest was a dynamic range limited using the Collewet method and quantized to 64 intensity levels, as in this work. The study investigated the predictive ability with local recurrence, distant metastasis, and disease-free survival as endpoints. They selected a least absolute shrinkage and selection operator (LASSO) model to preselect delta-radiomics texture features and used a Cox regression analysis to determine the prognostic ability of the features. Delta-radiomic features were predictive of all study outcomes. Interestingly, GLCM sum average and GLSZM large zone low gray-level emphasis were selected in their study as predictive. Only second order texture features were included for this work for a few reasons. The number of features was limited to keep the feature space small for the limited sample size of patients. The texture features included have been a mainstay in radiomics texture analysis studies, since their introduction and previous work conducted by the authors included repeatability analysis of the same features included in this work (24). Lastly, as the feature space begins to include higher order texture features, the interpretability of such features can become very difficult.

Fave’s work exposed the possibility of extracting predictive information from low-quality setup images and set the stage for applications in low field strength daily patient setup MR images. Boldrini et al. used 0.35-T setup images of rectal cancer patients acquired at fractions 1, 5, 10, 15, 20, and 25 to predict complete clinical response (16). Delta-radiomics features were defined as the ratio between pre-RT (fraction 1 setup image) subsequent time points. The most predictive timepoint was after the delivery of 22 Gy (physical dose). GLCM energy, GLRLM gray-level nonuniformity, and least axis length were selected as predictive of complete clinical response. Although only 16 patients were included, the selection of GLCM energy as one of the best radiomic predictors is encouraging.

Nasief et al. used CT-based delta-radiomics texture features extracted from pancreatic cancer patients treated with 28 fractions of CT-guided chemoradiation therapy to predict pathological response (22). Delta-radiomics texture features were calculated using the relative change in feature values from fraction 1 to each subsequent fraction. Comparisons of the current study and Nasief et al. should be minimized due to the differences in imaging modalities and fractionation, but Nasief et al. were able to achieve an AUC = 0.94 that is very encouraging of the performance of delta-radiomics texture features. A recent study by Cusumano et al. used 35 pancreatic cancer patients and considered radiomic texture features extracted from time points defined by delivered BEDs of 20, 40, and 60 Gy to predict local control following MR-guided RT (44). The patient library used for their work included patients treated with 5 fractions (17 patients) and 15 fractions (18 patients), and the use of BED was used to group patients to similar points in treatment. In our current study, the BED was used to group changes in features with delivered BED. Cusumano et al. extracted a total of 60 radiomic texture features that adhered to IBSI feature definitions. One feature calculated at the BED 40 Gy time point was predictive of local control at 1 year, with AUC = 0.78 and with 95% confidence interval of 0.61–0.94 but did not overlap with the features selected in this study. The large confidence interval could easily be attributed to the heterogeneity of fractionation of the patient population, and it is possible that different features could be prognostic at different points in time for different study endpoints. The pancreatic patients in this current work are all treated with SBRT, while Cusumano et al. used hypo-fractionated and SBRT patients. The results of this work suggested that changes induced from a baseline pre-RT value early in SBRT may have clinical utility. With both studies, the way response has been defined is likely an oversimplification of response, and the underlying biology and the accuracy of delta-radiomics feature analysis could be suppressed.

There are a few shortcomings of this study. Besides possibly oversimplifying response, the use of mRECIST for response determination is less than ideal, as it relies itself on imaging-based evaluation. Expansion of the library to include more patients with pathology-based responses could significantly strengthen results, as the response for 10 patients was determined with pathology (versus 20 using mRECIST) in this work. The development and further validation of more specific response evaluation methods, such as PET response criteria in solid tumors (PERCIST) and/or metabolic imaging, might help increase certainty of classifying patients as RS and NR (45). The relatively small sample size may not represent a fair sample of the true patient population, and a larger patient cohort is being pursued that will allow external validation. The use of the internal validation AUC in this study served its purpose, providing a common measuring stick to evaluate whether delta-radiomics texture analysis is feasible and could contain predictive utility. Although the behavior of the features in **Figure 3** is easy to see when graphed and analyzed, it is disconnected from any underlying changes to the tumor microenvironment. In addition, from **Table 3**, it is apparent that the time and range of dose delivered between the pre-RT (fraction 1 setup scan) and BED40 point compared to the BED20 point is spread out. Without correlation studies and larger patient cohorts, it is difficult to understand that the underpinnings GLCM sum average and NGTDM busyness are quantifying. This is a portion of research that radiomics texture features must bridge prior to wide clinical acceptance. The BED20 delta-radiomics texture features could be capturing and quantifying an initial inflammatory mechanism, a change in tumor signaling, or even just the altering of the physical cellular structure after radiation damage. It is possible that by the BED40 dataset’s acquisition, the delta-radiomics has become too heterogenous to extract any predictive features in the small cohort of 30 patients, or these mechanisms have simply stopped. Lastly, but always prevalent in radiomic texture analysis studies, variability in target volume contouring may impact results.

## Conclusion

This work sought to explore the application and feasibility of delta-radiomics texture analysis in low field strength daily MR setup images. While it remains to be seen if the predictive power of the delta-radiomic texture features selected and the method of analysis utilized in this study will hold in a larger patient cohort, the application of delta-radiomics should be further pursued. The workflow developed in this study could be easily modified and applied to larger data sets as a step in building clinical models and applied to other clinical sites. Changes detected early in treatment could provide valuable information with time left for physicians to consider changes to the treatment regime, including further dose escalation or alternative immunotherapies. Further expansion of the patient library using more patients, external validation, and exploration of a multi-institutional dataset for building a model would appear to be justified.

## Data Availability Statement

The datasets presented in this article are not readily available because the raw image data will not be shared due to ongoing data collection. Requests to access the datasets should be directed to Garrett Simpson, GNS24@miami.edu.

## Ethics Statement

The studies involving human participants were reviewed and approved by ViewRay Omnibus Patient Registry and Image Analysis Protocol, IRB#: 20160817, University of Miami IRB. The patients/participants provided their written informed consent to participate in this study.

## Author Contributions

GNS performed texture analysis and statistical analysis, drafted manuscript, and prepared the figures/tables. WJ gathered patient response data, verified contouring, and made manuscript contributions. BS gathered patient response data, verified contouring, and made manuscript contributions. LP gathered patient response data, verified contouring, and made manuscript contributions. EM verified patient contouring and made manuscript contributions. DK was the statistician who contributed to machine learning work. JCF contributed to texture analysis, methods, figures, tables, and manuscript sections. ND contributed to texture analysis, methods, figures, tables, and manuscript sections. All authors contributed to the article and approved the submitted version.

## Conflict of Interest

The authors declare that the research was conducted in the absence of any commercial or financial relationships that could be construed as a potential conflict of interest.

## Publisher’s Note

All claims expressed in this article are solely those of the authors and do not necessarily represent those of their affiliated organizations, or those of the publisher, the editors and the reviewers. Any product that may be evaluated in this article, or claim that may be made by its manufacturer, is not guaranteed or endorsed by the publisher.
